# Does Primary Narcissism Exist in Newborn Babies? Evidence from Sleep Science

**DOI:** 10.3389/fpsyg.2012.00330

**Published:** 2012-09-07

**Authors:** Lampros Perogamvros

**Affiliations:** ^1^Division of Neuropsychiatry, Department of Psychiatry, University Hospitals of GenevaGeneva, Switzerland; ^2^Department of Neuroscience, University of GenevaGeneva, Switzerland

The term *narcissism*, influenced by the Greek myth of Narcissus, refers to self-love. According to Freud (1914, p. 73–74), newborn babies are characterized by primary narcissism, that he defines as the “*libidinal complement to the egoism of the instinct of self-preservation*.” In other words, primary narcissism, which would predominate until the development of the Ego, corresponds to an innate instinctual behavior which is mainly driven by the desire and energy of the newborn to survive, and by a capability of satisfying these instincts on itself (“*auto-erotism*”). This early period of human's life has been described as “*objectless*” (Tausk, 1919), because the external objects with their potential unpleasant effects are excluded from baby's perceptions and interest (Ferenczi, 1909). Instead, it is the newborn's own body and own organs that are considered as *love-objects* (Freud, 1911, p. 60). Later on, a part of this *libidinal* mental and emotional energy investment would be redirected onto external objects, creating an opposition between *Ego-libido* and *Object-libido*. To date, no empirical or other scientific evidence has confirmed the existence of primary narcissism. Here, I will try to show how recent findings in sleep science offer similarities between newborn's sleep state and primary narcissism.

From a theoretical point of view, early and more recent writings draw a link between sleep and primary narcissism. Freud argues that sleep represents a stage of unconscious primary narcissism and that, even after the development of the Ego, sleep will stay as a period of withdrawal toward this narcissism (Freud, 1917, p. 222–223). During sleep, the person would return to a developmental starting point, reliving what the fetus experienced in the maternal body. Interestingly, almost one century later, Jaak Panksepp claimed that an archaic affective state is expressed in rapid-eye movement (REM) sleep (Panksepp, 1998, p. 134–135). These authors orient our research concerning the existence or absence of primary narcissism, toward sleep. Recent and older findings in sleep science may help us clarify the unconfirmed and debated question of primary narcissism in newborns.

Empirical and experimental studies offer us more insight into how sleep and the Self interact in newborns. Since as early as the 13th week of gestation, smiles and other facial expressions are extremely common (Kurjak et al., 2005; Yigiter and Kavak, 2006) and happen almost exclusively during active sleep (REM sleep) and never or very rarely while the infant is fully awake or in quiet sleep (NREM sleep; Dondi et al., 2007). Originally thought to be related to digestion (older literature may refer to them as vegetative or gas smiles), it was later suggested that, due to their preservation in infants with microcephaly, neonatal smiles are caused by subcortical activation and that they occur in the absence of recognized external or internal (visceral) stimuli (*endogenous smiling system*; Emde and Koenig, 1969). More recent articles also claimed that these expressions are not related to social experience and had no relationship to cortically driven positive affect (Messinger and Fogel, 2007). Actually, social smiling is not present at birth and emerges much later (at about 2 months of age; Dondi et al., 2007). It depends on neurological maturity, infant attention and maternal expressions and, from an evolutionary point of view, it would help keep parents and other potential caregivers close at hand (Bowlby, 1982).

What would then be the functional role of emotional expressions while the newborn sleeps and is thus typically deprived of interactions with its environment? It is now established that REM sleep and dreaming have dissociated mechanisms, with a brainstem mechanism being responsible for REM sleep and a forebrain mechanism for dreaming (Solms, 2000). In addition, REM sleep occurs much earlier in human development than dreaming, by occupying the biggest and first stage of newborn's sleep (Hobson, 2009). Its great prominence during early life would assist the process of central nervous system maturation and differentiation (Marks et al., 1995). Emotional expressions during REM sleep of the infant would then be related to necessary and primordial learning processes (Fifer et al., 2010), adequate emotional reactions (Dondi et al., 2007; Berger et al., 2012), and to the long-term consolidation of emotional memories, in a similar way as in adults (Sterpenich et al., 2009). Recent neuroimaging studies support this claim by showing that, similarly to the correlation between REM sleep and limbic activation in adults (Maquet et al., 1996), an early functional specialization of emotions during sleep takes place in infants. For example, modulation of brain regions related in emotional processing, such as the orbitofrontal cortex and insula takes place in response to emotional vocalizations during sleep (Blasi et al., 2011).

Quiet sleep seems also important for the emotional, cognitive, and sensorimotor maturity of human. It was recently shown that a normal shift between quiet sleep and wakefulness (contrary to rapid cycles between states of high arousal, such as active sleep and cry) is important for the optimal emotional and cognitive development, for less negative emotionality and better symbolic and executive competences at 5 years (Weisman et al., 2011). In addition, delta-brushes (a dominant pattern of rapid oscillatory activity in quiet sleep of human neonates) triggered by spontaneous fetal movements *in utero* or in newborns, seem to be responsible for the formation of cortical connections required for sensorimotor coordination and body representation (Milh et al., 2007). Importantly, this process takes place before the brain starts to receive sensory input from the external world (Milh et al., 2007). Finally, it has been proposed that an activation of mesolimbic reward and instinctual exploratory motivational networks (SEEKING system) is present during sleep in mammals (Perogamvros and Schwartz, 2012). This activation would be related to memory consolidation, learning enhancement, as well as to dream generation, and its motivated content (Solms, 2000; Perogamvros and Schwartz, 2012).

All the aforementioned recent studies suggest that networks of critical significance for the individual's survival and development (“*instinctual drives for self-preservation*”) are (1) activated in the infant's sleep, and (2) precede and contribute to the development of cortical networks necessary for later-in-life sensorimotor, mental, and emotional interaction with the external environment (“*objects*”). Importantly, the newborn's reactions (positive or negative) to stimuli, if present, mainly concern an emotional and cognitive “*internalization*” during sleep (are thus “*objectless*”). More specifically, the aforementioned emotional/motivational (“*libidinal*”) networks subserve necessary self-oriented sleep-specific, sensorimotor, cognitive, and emotional processes during a phase where the person is typically deprived of external feedback (sleep) and is thus protected from unpleasant origins of frustration that he or she cannot control (what would strengthen the feeling of “*omnipotence*”). Considerably, these processes contribute to the formation of cortical connections, emotional maturity, and waking consciousness, in a totally secure environment like sleep. Therefore, I claim that the activation of these sleep processes, which at that stage of life massively serve the development of the emotional Self and the bodily Self (“*body and its functions as* *love-objects*”), offers striking similarities with primary narcissism and its developmental necessity in newborn babies, as proposed by Freud. In accordance with Freud's assumption (Freud, 1920, p. 360), sleep would represent the *archetypical* state of primary narcissism because of these specific off-line emotional/motivational attributes and its predominance in early life (16–17 h per day). When the cortical Self-awareness (“*Ego*”*)* develops later on during infancy, the child will start to sleep less (10–12 h per day) and to interact non-instinctually with its environment during waking life (“*externalization*”). During that phase (individuation-separation phase of child development, according to Mahler, 1967), the child will start understanding how the environment is differentiated from the Self, which will gradually lose its “magic powers” (it will always keep them during sleep though). The emotional/motivational (“*libidinal*”) drives could be now potentially addressed toward the environment (“*Object-libido*”), and be confronted with it.
